# Effects of maternal androgens and their metabolite etiocholanolone on prenatal development in birds

**DOI:** 10.1242/jeb.247205

**Published:** 2024-08-05

**Authors:** Yuqi Wang, Bernd Riedstra, Ton Groothuis

**Affiliations:** University of Groningen, Groningen Institute for Evolutionary Life Sciences, 9700 AB Groningen, The Netherlands

**Keywords:** Embryonic heart rate, Testosterone, Embryonic metabolism, Androstenedione, Maternal effects, Embryonic hormone production

## Abstract

Offspring phenotypes can be affected by maternal testosterone and androstenedione (A4), which are considered a tool of mothers to adjust offspring to a fluctuating environment. Yet testosterone and A4 are very rapidly metabolized by developing avian embryos, suggesting that either the maternal testosterone and A4 have potent organizational effects on the embryos extremely early before being metabolized or it is the metabolites that evoke phenotypic variation in the offspring. One of the metabolites, etiocholanolone, increases substantially during early embryonic development and is a likely candidate for mediating maternal effects as it can promote erythropoiesis. To investigate and compare the effects of testosterone and A4 with the possible effects of etiocholanolone during prenatal embryonic development, we increased their levels in black-headed gull eggs (*Larus ridibundus*), and used sham-injected eggs as controls. This species usually has 3-egg clutches in which maternal androgen levels increase with the egg-laying sequence. We analysed embryonic heart rate, peri-hatching biometric traits, the ratio of white to red blood cells (W/R ratio) and bursa development. We found that testosterone and A4 treatment increased embryonic heart rate irrespective of egg-laying sequence and decreased bill length and W/R ratio, whereas etiocholanolone did not mimic these effects. Instead, etiocholanolone treatment decreased tarsus length and brain mass. Our finding that etiocholanolone does not mimic the effects induced by testosterone and A4 suggests that the embryonic metabolism of maternal testosterone and A4 can potentially diversify the function of these maternal androgens.

## Teaser

**Summary:** Bird embryos modify hormones received from the mother in the egg and thereby affect their own development.

## INTRODUCTION

In many oviparous species, substantial amount of androgens from maternal origin, such as androstenedione (A4) and testosterone, are present in the yolks of freshly laid eggs (Groothuis et al., 2005). Studies have shown that maternal androgens can vary systematically with laying order or environmental factors and that they have widespread effects on offspring development (von Engelhardt and Groothuis, 2011). The differential allocation of maternal androgens is considered a tool of the mothers to adjust the development outcomes of the offspring to the prevailing current environmental conditions (Groothuis et al., 2005; Groothuis and Schwabl, 2008).

However, recent studies have revealed that the maternal androgens are rapidly metabolized by the embryos at very early developmental stages into metabolites that are either believed to be less biologically active or whose biological functions are not well known (Kumar et al., 2018; Campbell et al., 2020). This raises the question: how can maternal androgens have such potent effects on offspring development despite being so rapidly metabolized by the embryos? One possible explanation is that the maternal androgens exert their organizational effects on the embryos' developmental trajectory before being metabolized (Phoenix et al., 1959; Arnold, 2009). Alternatively, it could be the metabolites that mediate the phenotypic variation in the offspring. Intriguingly, the metabolism of maternal hormones, and thereby their effects, are at least partly under embryonic control (Kumar et al., 2019b; Wang et al., 2023c).

Etiocholanolone (ETIO) is one such metabolite that can be metabolized from testosterone and A4 via different catalysing pathways and is an end product in the androgen metabolism pathway (Kaminski et al., 2005). Recent studies showed that egg ETIO levels increase substantially within the first few days of incubation while testosterone and A4 levels decrease (rock pigeon C*olumba livia*: Kumar et al., 2018; Wang et al., 2023b; European starling *Sturnus vulgaris*: Campbell et al., 2020). As a result of the addition of hydroxy groups during metabolism, ETIO is more hydrophilic than testosterone and can therefore be more easily taken up by the embryo from the yolk into its circulation, which further supports the possibility that this hormone is involved in affecting offspring phenotypic variation (Paitz and Bowden, 2011).

ETIO has been suggested to promote erythropoiesis during embryonic development (birds: Levere et al., 1967; Irving et al., 1976; mammals: Gordon et al., 1970), by inducing haemopoietic stem cell differentiation into erythroid cells (Gordon et al., 1970; Cumano and Godin, 2007). As erythrocytes play a critical role in carrying oxygen and therefore its abundance could influence embryo metabolic rate, it is reasonable to assume that increasing the level of ETIO promotes prenatal embryonic development. Interestingly, a boost in post-hatching growth is one of the commonly found effects of exposure to elevated maternal testosterone (reviewed by von Engelhardt and Groothuis, 2011). It is thought that this effect could be due either to a shortened incubation time till hatching, getting a head start over siblings, or to increased or more vigorous begging for parental food provisioning, both of which are known effects of high prenatal maternal testosterone exposure (Eising et al., 2001). To our knowledge, only one study in European starlings assessed the effects of exogenous ETIO on embryonic growth *in ovo*, but reported no effect on total tissue mass (Campbell et al., 2020).

Increased prenatal testosterone exposure has also been found to cause immunosuppression (Groothuis et al., 2005; Muriel et al., 2015; Tobler and Sandell, 2009; Müller et al., 2005), including decreased leukocyte count (Al-afaleq and Homeida, 1998; Puerta et al., 1995; Nava et al., 2001). This may be linked to ETIO as well, as the boosting effect of ETIO on erythropoiesis might be traded off against lymphopoiesis (e.g. Andersson et al., 2004), as both types of blood cells are continually produced from a pool of progenitors in the yolk sac or later from the embryo liver and spleen (Golub and Cumano, 2013). This has as yet not been tested.

To investigate whether the maternal androgens (i.e. testosterone and A4) directly or their metabolites indirectly mediate the observed maternal effects, we manipulated testosterone and A4 or ETIO in freshly laid eggs of black-headed gulls (*Chroicocephalus ridibundus*). Black-headed gulls were chosen as our study species because the post-hatching hormone-mediated maternal effects are well studied in this species in terms of hatching time, growth, immunity and behaviour (Eising et al., 2001; 2003; Groothuis et al., 2005; Müller et al., 2005). However, a hormone metabolism profile from the early embryos of this species is still lacking. We therefore first analysed and quantified maternal androgen metabolism in the eggs of black-headed gulls and, based on this profile, we treated eggs with either a combination of A4 and testosterone, or ETIO, or vehicle (see below), and investigated whether there were differences in (1) the rate of embryonic metabolism by measuring heart rate (Sheldon and Griffith, 2018; Wang et al., 2023a) during mid to end incubation, (2) growth in terms of brain, heart and liver mass, head–bill and tarsus length, (3) the ratio of circulating white to red blood cells (W/R ratio), and (4) bursa development shortly before hatching (day 18 of incubation). We predicted that increased A4 and testosterone levels increase embryonic heart rate, boost growth and decrease W/R ratio. As one of the main metabolites of maternal androgens, we further investigated to what extent ETIO mimics these effects.

## MATERIALS AND METHODS

### Animal housing

Forty pairs of black-headed gulls, *Chroicocephalus ridibundus* (Linnaeus 1766), were housed in an outdoor aviary (dimensions: 45 m length×9.6 m width×3.75 m height) with two shallow water ponds under natural light and temperature and with *ad libitum* access to food and fresh water. Nesting materials were provided in the aviary. Daily observations were made for food and water availability, nest building and egg laying. All animal procedures were approved by the animal welfare committee of the University of Groningen and carried out under the guidelines of the committee.

### Egg collection and incubation

Eggs were collected from the outdoor aviary of the University of Groningen in 2020 and 2022, and from a wild black-headed gull colony situated on an inland island of The Netherlands in 2021 and 2022. Black-headed gulls usually produce 3-egg clutches on consecutive days with a laying interval of 2 days (Weidmann, 1956; Müller et al., 2004). The nests in the outdoor aviary were visited between 11:00 h and 12:00 h each day. Freshly laid 1st, 2nd and 3rd eggs were collected. The nests of the wild colony were checked in early May, and eggs were collected when the nest contained only one cold egg as 1st laid eggs, as gulls start to incubate only when the 2nd and 3rd eggs are laid. All collected eggs were marked with a unique egg identity and then transported to our indoor facility, where the eggs were weighed to the nearest 0.1 g. The eggs were randomly assigned to one of the treatment groups (see below, Table 1; see also Table S1). After treatment, eggs were placed in an incubator with a setup of 37.4°C, 55% relative humidity and automatic egg turning every 12 h (Ova-Easy Advance Series II Digital Cabinet Egg Incubator, Brinsea Products Inc., Titusville, FL, USA). The eggs were monitored for survival by monitoring heart rate (see below) until day 18 of incubation when all eggs were terminated by placing the eggs at −20°C. The sample size over treatments and egg-laying sequence is shown in Table S1. There was no treatment or egg-laying sequence effect on survival until day 18 of incubation (ANOVA, both *F*<2.31, *P*>0.10).

**Table 1.**
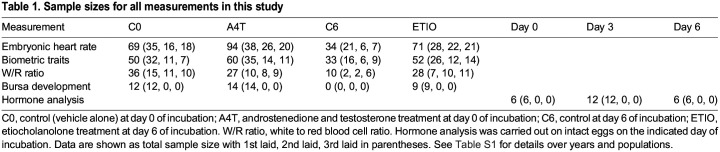
Sample sizes for all measurements in this study

### Hormone analysis

A separate egg collection (1st laid eggs only, *n*=24, Table 1) was made for hormone analysis, assessing the changes in hormones over the first 6 days of incubation. Hormone extractions and analyses followed Kumar et al. (2018). In brief, the eggs were thawed at room temperature, and the yolk and the albumen were homogenized and used for hormone extractions. For each egg, 300 mg of the homogenate was used for liquid chromatography combined with tandem mass spectrometry (LC-MS/MS) to determine the level of testosterone, conjugated testosterone and A4 (conjugated androstenedione was excluded as a target because it lacks a free hydroxyl group in its structure and hence cannot be conjugated). Meanwhile, 600 mg of the homogenate was used for gas chromatography combined with tandem mass spectrometry (GC-MS/MS) to determine the level of ETIO and conjugated ETIO. Internal standard (LC-MS/MS: 25 μl of 30 nmol l^−1 13^C_3_-labelled testosterone in 50% methanol; GC-MS/MS: 100 μl: of 6.7 µmol l^−1 2^H_5_-labelled etiocholanolone in 100% methanol; both from IsoSciences, Ambler, PA, USA) was added to the samples, mixed thoroughly and left for 1 h at room temperature for equilibration. Then, each sample was extracted twice in 1 ml methanol. The supernatant was then transferred to tubes containing solid ZnCl_2_ (200 mg for LC-MS/MS; 300 mg for GC-MS/MS) for lipid precipitation. The final eluate was obtained through C18 columns (#5138775, Aurora Borealis, Schoonebeek, The Netherlands) for LC-MS/MS or HLB cartridges (#WAT094226, Waters Chromatography BV, Etten-Leur, The Netherlands) for GC-MS/MS. The eluate was then divided into two equal parts, either with or without hydrolysis (to acquire free or free and conjugated hormones), and transferred to University Medical Center Groningen for chromatographic separation and mass spectrometry.

### Hormone solutions

**Fig. 1.**
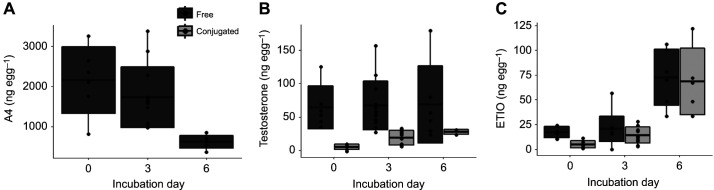
**Hormone profiles for 1st laid eggs at day 0, 3 and 6 of incubation.** (A) Androstenedione (A4), (B) testosterone and (C) etiocholanolone (ETIO) in free and conjugated forms. Boxplots show means (middle bar), s.d. (hinges) and minimum–maximum values (whiskers).

Each egg received 50 µl of one of the following hormone solutions (with sesame oil as vehicle solvent): androstenedione and testosterone combination (A4T), etiocholanolone (ETIO) or vehicle-only control. A4T solution was made to increase androstenedione and testosterone levels by two times their standard deviation (s.d.) in 1st laid eggs at oviposition. The final A4T solution had 33,205.6 ng ml^−1^ A4 (increase of 1660.3 ng A4 per egg) and 511.6 ng ml^−1^ testosterone (increase of 25.6 ng testosterone per egg) (Müller et al., 2004; Kumar et al., 2019a). The ETIO solutions were made to increase its level by two times its s.d. in the 1st laid egg on day 6 of incubation, as the hormone analysis showed that ETIO substantially increased on this day (Fig. 1C) and blood island formation in bird embryos starts around day 7 of incubation (Ferkowicz and Yoder, 2005; Moore and Metcalf, 1970). The final ETIO solution had 1140.8 ng ml^−1^ ETIO (increase of 57.0 ng ETIO per egg).

### Hormone treatments

Eggs were randomly assigned to one of four treatment groups. The C0 and A4T groups were injected on day 0 of incubation with control or A4T solution, respectively. The C6 and ETIO groups were injected on day 6 of incubation with control or ETIO solution, respectively. Injecting ETIO on day 6 of incubation aimed to mirror the timing of its increase in intact eggs (as shown in Fig. 1C) and the C6 group was set up to control the potential effects of manipulation time. Eggs were placed horizontally in an egg holder for 5 min to allow the yolk to float to the upper side. The eggshell was wiped with a cotton pad containing 75% ethanol for disinfection and a small hole was drilled in the eggshell (on the top side, approximately 2 mm from the central axis, and approximately two-thirds of the way toward the air chamber). Then, a disposable insulin syringe (U-100, 29G needle×12.7 mm, BD Micro-Fine, Le Pont-de-Claix, France) was inserted through the hole to inject one of the hormone solutions (see above). After injection, a drop of Vetbond (3M, USA) was applied to seal the puncture spot on the eggshell.

### Embryonic heart rate monitoring

Embryonic heart rates were detectable by using an electronic infrared monitor (Buddy, Vetronic Services, Abbotskerswell, Newton Abbot, UK) from 8 days after the onset of incubation onwards (the Buddy gives inconsistent readout before day 8 of incubation). Eggs from day 8 to day 18 of incubation were temporarily removed from the incubator every day between 12:00 h and 13:00 h and heart rate measurements were obtained within a 3 min period, as previous studies observed no obvious decrease in heart rate due to cooling in this time frame (Lierz et al., 2006). Immediately after the heart rate was determined, eggshell temperature (referred to as egg temperature hereafter) was measured using an infrared thermometer (Welquic WI-03) as the heart rate of embryos is strongly influenced by temperature. If the heart rate did not stabilize within 3 min, eggs were put back into the incubator and we retried 1 h later.

### Blood sampling and haemogram measurement

In 2020 and 2022, eggs that had been incubated for 18 days were removed from the incubator and placed horizontally in an egg holder. A section of eggshell and inner membrane approximately 10 mm width×10 mm length was removed with tweezers, a blood vessel was isolated and punctured by a needle. Whole blood was drawn by a red blood cell-diluting pipette (1:200, witeg Labortechnik GmbH, Wertheim, Germany) followed by drawing Natt and Herrick's solution (Campbell, 2015) to stain blood cells and dilute the whole blood 200 times. The diluted blood was discharged into a haemocytometer counting chamber (Neubauer Improved Haemocytometer Counting Chamber, BD, Heidelberg, Germany) and examined under a microscope (400x magnification) for the number of different types of cells (Campbell, 2015). The concentration of erythrocytes and leukocytes in the whole blood was then calculated following Fudge (2000).

### Biometric measurements

The embryonic brain, heart and liver were dissected. Wet mass of the brain, the heart, the liver and the rest of the body were obtained to the nearest 0.001 g. The tarsus length and beak length of the embryos were determined using digital photography with ImageJ (v.2.0.0-rc-59/1.51k) following Rueden et al. (2017) and Williams et al. (2020).

### Bursa analysis

Bursa analysis was carried out in 2021 on 18 day old embryos (*n*=35) (Table 1). The body trunks containing the bursa were placed in a 10% formalin solution overnight for immunohistochemistry analysis. The bursas were dissected out of the body trunks and these formalin-fixed specimens were then embedded in paraffin, sectioned (4 µm thick) and stained with haematoxylin and eosin according to the method described by Aihara et al. (2015). Immunohistochemistry (IHC) staining of preliminary B lymphocyte marker CD79a (Mason et al., 1995; Sayegh et al., 2000) was carried out by the Royal GD animal health service lab (Deventer, The Netherlands).

### Sexing the embryos

A blood sample from each embryo was taken for molecular sex determination following Griffiths et al. (1998). DNA was extracted with a standard ammonium acetate protocol (Qamar et al., 2017). A standard PCR protocol was used to amplify the sex-linked *CHD* gene, using primer pair 2602F/2669R (2602F: 5′CAGATGGTGAGGATGCTGGAC3′ and 2669R: 5′CCCTTTTATTGATCCATCAAGYCTCTRAAGAG3′; van der Velde et al., 2017). PCR products were separated on 2% agarose gels stained with ethidium bromide and scored visually; females showed two bands (W-linked and Z-linked copies of the *CHD* gene), whereas males showed only a single band (Z-linked only).

### Statistical analysis

Changes in hormone levels due to metabolism over the first 6 days of incubation were tested by linear mixed models (LMMs), with each hormone separately as a response variable, incubation day as the independent variable and nest identity as a random effect.

To address our specific hypotheses on the effects of the different hormones on embryonic traits, statistical analyses on the effect of treatment were performed to compare: (1) C0 and A4T eggs in order to test the hypothesis that A4 and/or testosterone affect metabolism and growth; (2) C6 and ETIO eggs to test whether ETIO itself affects embryonic development; and (3) A4T and ETIO eggs, to test the hypothesis that ETIO mimics at least part of the effects of the primary androgens, or has other functions. As the effect of year is confounded with egg-laying sequence (only 1st laid eggs were collected in 2021) and we do not have a hypothesis about year effects, we left ‘year’ out in our analyses.

To describe and analyse the non-linear patterns of embryonic heart rate throughout the incubation period, a set of generalized additive models (GAMs) were generated following Wang et al. (2023a). Based on the whole dataset, a null model was set with heart rate as the response variable, egg identity as random intercept and mean-centred eggshell temperature as covariate (as it is positively related to embryonic heart rate, Pearson correlation, *P*<0.001; see also Sheldon and Griffith, 2018). Then, a set of models (see Table S2) was constructed with or without treatment, egg-laying sequence and their interaction as intercept(s). Smoothing function(s) for the age of the embryos was included to model the changes in heart rate curves and was allowed to vary by treatment, egg sequence or their interaction. Additive effects of treatment and egg-laying sequence but not their interaction (because of low statistical power) were also tested for the intercept and curve shape. Given that the heart rate should be zero at the onset of incubation and it was detectable to us only from 8 days of incubation and onwards, and to make the intercept meaningful for extrapolation, the values of embryo age were subtracted by 8 units, such that day 8 of age received the value zero, 9 became 1 and so on. From this set of GAMs, the optimal model formula was selected also following Wang et al. (2023a). To address our hypotheses, three GAMs with the optimal model formula were used to compare embryonic heart rates between different treatments.

Multivariate analysis of covariance (MANCOVA) was used to analyse the effect of treatment on embryo biometric traits, as wet mass of the brain, heart, liver and the remaining carcass and length of the beak and tarsus at day 18 were significantly correlated linearly (Table S3). Wet brain mass, wet heart mass, wet liver mass, tarsus length and beak length were used as response variables, and treatment, egg-laying sequence and their interaction were included as predictors. Embryo sex and egg mass at oviposition were used as covariates. Nest identity was set as a random intercept. Next, univariate tests were performed for each embryonic trait. Effect sizes (Cohen's *d*) of the treatment on embryo morphometric traits and body composition were estimated from marginal means.

To analyse the effects of treatments on embryo blood cell composition, LMMs were used with the ratio of blood leukocyte to erythrocyte count (hereafter W/R ratio) as a response variable, treatment, egg-laying sequence and their interaction as predictors, embryo sex as a covariate, and nest identity as a random effect. As C0 and C6 eggs showed no differences in either erythrocyte or leukocyte count (LMM, *F*=2.52, *P*=0.13), they were merged as control eggs to increase statistical power. Tukey tests were used as *post hoc* tests to analyse the different effects of treatment depending on egg-laying sequence.

Fisher's exact tests were used (because of the small sample size) to analyse the effects of treatments on the abundance of CD79a-positive cells in the bursa.

## RESULTS

### Hormone metabolism profile

A4 decreased with incubation duration (*F*=8.25, *P*=0.002; Fig. 1A), while testosterone remained unchanged (*F*=0.02, *P*=0.98; Fig. 1B). Conjugated testosterone (*F*=11.23, *P*<0.001; Fig. 1B), ETIO (*F*=22.16, *P*<0.001; Fig. 1C) and conjugated ETIO (*F*=23.81, *P*<0.001; Fig. 1C) increased with incubation duration (see also Table S4).

### Effects on embryonic heart rate

**Fig. 2.**
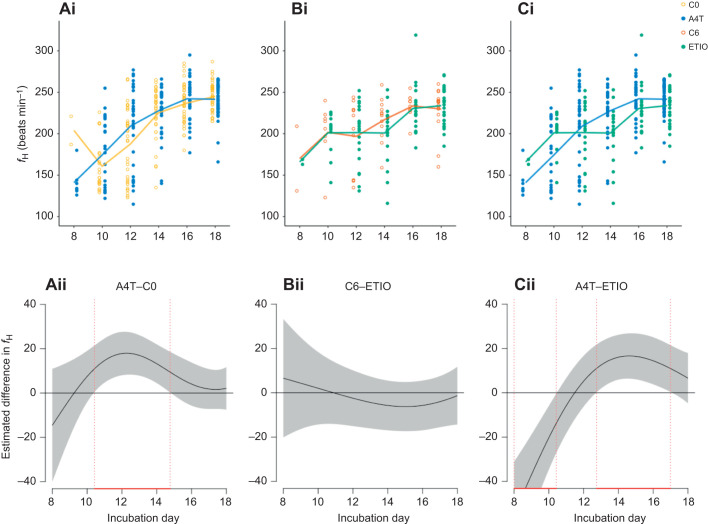
**Embryonic heart rate from different treatment groups during incubation.** (Ai–Ci) Raw data and mean values of heart rate (*f*_H_) for each treatment group. (Aii–Cii) Pairwise comparisons of estimated heart rate differences between eggs generated from generalized additive models (GAMs). The shaded area indicates the 95% pointwise confidence interval. The time window showing a significant difference during incubation is indicated by red dotted lines. C0, control (vehicle) at day 0 of incubation; A4T, androstenedione and testosterone treatment at day 0 of incubation; C6, control at day 6 of incubation; ETIO, etiocholanolone treatment at day 6 of incubation.

Embryonic heart rate increased from day 8 to day 18 of incubation under all treatments (estimate=13.4, *F*=33.5, *P*<0.001; Fig. 2) but its pattern differed among treatments. Indeed, the top-ranked model from the GAM set showed that the variation in the increase of embryonic heart rate over incubation duration was best predicted by both treatment and egg-laying sequence as intercept and both treatment and egg-laying sequence as the curve shape (Table 2), without their interaction effect. A4T-treated embryos had higher heart rate than C0 embryos, especially between day 11 and 15 (estimate=8.09, *F*=5.78, *P*=0.02; Fig. 2Aii) but ETIO embryos showed no overall difference from C6 embryos (estimate=−2.29, *F*=0.11 *P*=0.74; Fig. 2Bii) or from A4T embryos (estimate=−3.83, *F*=0.66, *P*=0.42; Fig. 2C). Nevertheless, ETIO embryos had higher heart rate from day 8 to day 10 of incubation yet lower heart rate during day 13 and day 17 of incubation than A4T embryos (Fig. 2Cii). Egg-laying sequence showed no effect in all three comparisons (all *F*<1.13, *P*>0.32).

**Table 2.**
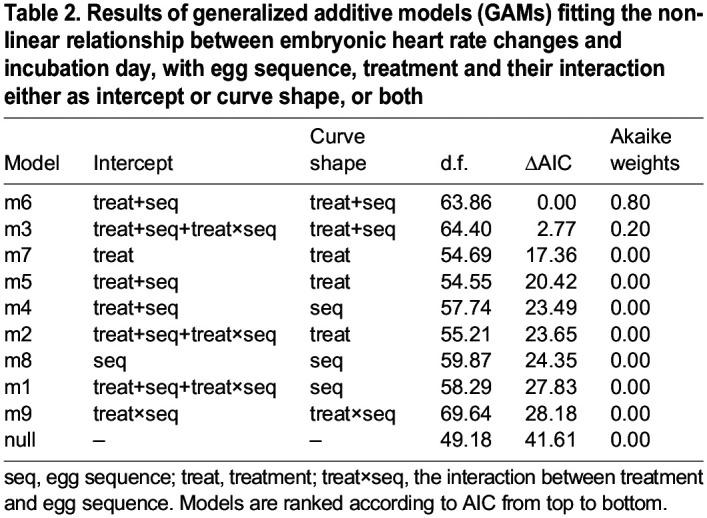
Results of generalized additive models (GAMs) fitting the non-linear relationship between embryonic heart rate changes and incubation day, with egg sequence, treatment and their interaction either as intercept or curve shape, or both

### Effects on biometric traits

Contrary to our expectation, there was in the multivariate test no overall effect of A4T treatment on the embryos' biometric traits compared with control embryos (*F*=1.74, d.f.=6, *P*=0.13). However, there was a negative effect of ETIO treatment (*F*=2.26, d.f.=6, *P*=0.05). Egg-laying sequence, the interaction between egg-laying sequence and treatment, egg mass at oviposition and embryo sex also did not affect the biometric traits (all *F*<1.34, d.f.=6, *P*>0.26). However, univariate tests showed that A4T embryos had a decreased beak length (*F*=6.20, *P*=0.02, Cohen's *d*=0.59, 1−β=0.99), whereas ETIO embryos showed a decreased tarsus length (*F*=5.92, *P*=0.02, Cohen's *d*=0.68, 1−β=0.99) and brain mass (*F*=8.99, *P*=0.004, Cohen's *d*=0.50, 1−β=0.98), compared with control embryos.

Interestingly, there was in the MANOVA an overall difference between the A4T and ETIO treatment on the embryos' biometric traits (*F*=2.36, d.f.=6, *P*=0.04). Univariate tests showed that ETIO embryos had a shorter tarsus length than A4T embryos (*F*=11.2, *P*=0.001, Cohen's *d*=0.71, 1−β=0.99), but there were no statistical differences between other biometric traits (Table 3; Table S5). It should be noted that for the majority of tests the statistical power was low.

**Table 3.**
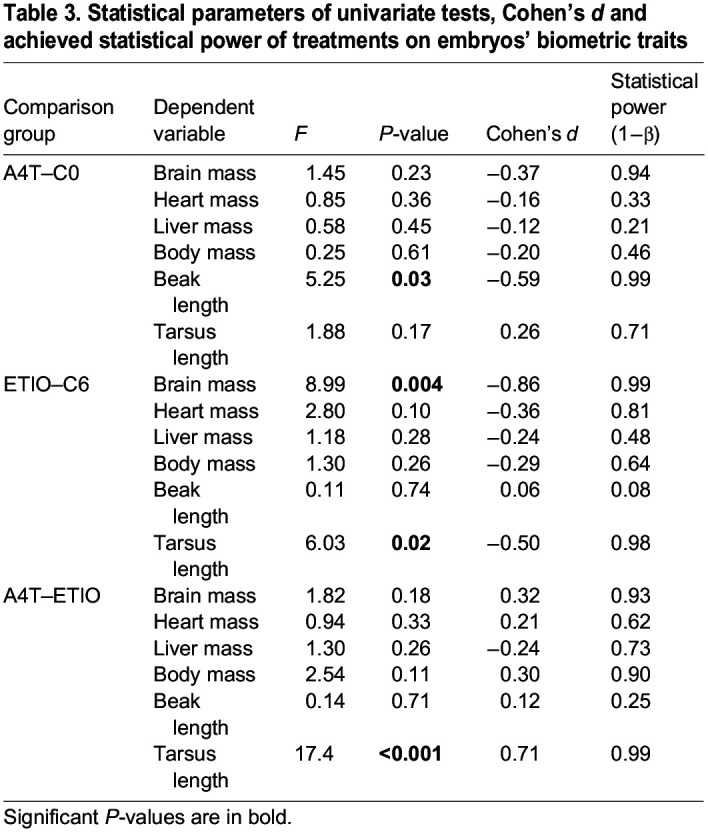
Statistical parameters of univariate tests, Cohen's *d* and achieved statistical power of treatments on embryos' biometric traits

### Effects on W/R ratio

**Fig. 3.**
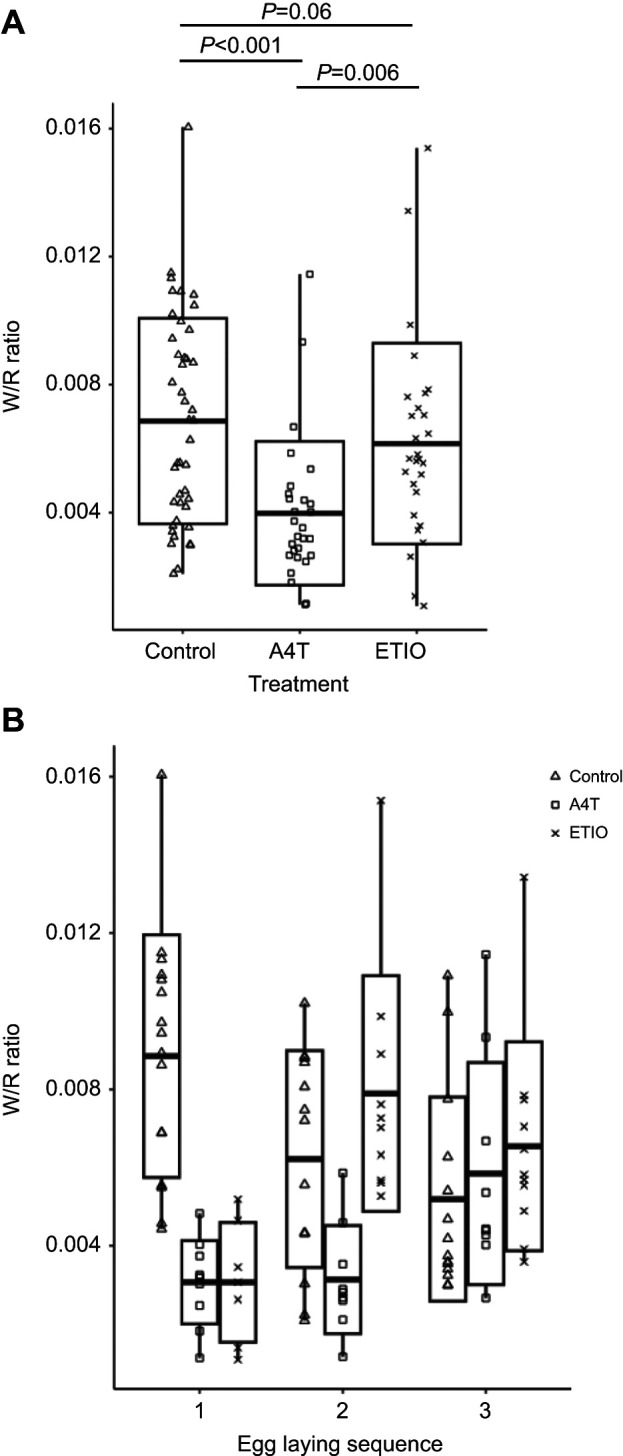
**White to red blood cell ratio (W/R ratio) of 18 day old embryos.** (A) W/R ratio for different treatment groups. (B) Treatment effects over different egg-laying sequences. Boxplots show means and s.d. with whiskers showing lower and upper values. Linear mixed model, *post hoc* Tukey's test. See Fig. 1 for a description of the treatments.

As expected, A4T embryos showed lower W/R ratio than control embryos (*F*=12.6, *P*<0.001; Fig. 3A), with no effect of egg-laying sequence or the interaction between treatment and egg-laying sequence (both *F*<1.60, *P*>0.21). Contrary to our expectation, ETIO embryos showed no overall W/R ratio difference from control embryos (*F*=3.80, *P*=0.06; Fig. 3A), There was also no effect of egg-laying sequence (*F*=0.51, *P*=0.60), but there was an effect of the interaction between treatment and egg-laying sequence (*F*=3.29, *P*=0.04, Fig. 3B). *Post hoc* tests showed that ETIO treatment only decreased W/R ratio in 1st laid eggs (Tukey, *t*=2.44, *P*=0.02) but not in 2nd laid (*t*=−0.85, *P*=0.40) or 3rd laid eggs (*t*=1.86, *P*=0.17; Table S6). Finally, A4T embryos showed overall lower W/R ratio than ETIO embryos (*F*=8.40, *P*=0.006; Fig. 3A), with an effect of both egg-laying sequence and the interaction between treatment and egg-laying sequence (both *F*>6.18, *P*<0.004). *Post hoc* tests showed that A4T embryos had a decreased W/R ratio compared with ETIO embryos only in 2nd laid eggs (Tukey, *t*=−4.43, *P*<0.001; Fig. 3B) but not in 1st laid (*t*=0.03, *P*=0.97) or 3rd laid eggs (*t*=−0.69, *P*=0.50; Table S6). There were no differences between the sexes in any test (all *F*<3.16, *P*>0.08).

### Effects on bursa

Bursa from all treatment groups at day 18 of incubation showed no signs of lesions and were only scarcely populated with CD79a^+^ cells. The abundance of CD79a^+^ cells did not differ among control, A4T and ETIO embryos (all *P*>0.64).

## DISCUSSION

This study tested to what extent ETIO, which has been shown to be produced in embryos by conversion of the maternal hormones testosterone and A4 in eggs of other avian species, affects embryonic development. To this end, we manipulated either ETIO or a combination of A4 and testosterone (A4T) at the time points at which they reach their peak levels during early embryonic development. In the first descriptive part of the study, we found that young black-headed gull embryos are able to substantially convert A4 and testosterone to conjugated androgens and ETIO similar to other avian species (e.g. Kumar et al., 2018; Campbell et al., 2020). In the subsequent experiment, we found that increasing A4 and testosterone in freshly laid eggs, mimicking maternal hormone deposition, resulted in increased embryonic heart rate, no change in overall prenatal growth, decreased W/R ratio and no change in CD79a+ cell abundance in the bursa. Yet, increasing ETIO, i.e. one of the embryonic metabolites of the maternal androgens, did not lead to similar effects (summarized in Table 4), with no change in embryonic heart rate, decreased overall prenatal growth, no change in W/R ratio and no change in CD79a^+^ cell abundance in the bursa.

**Table 4.**
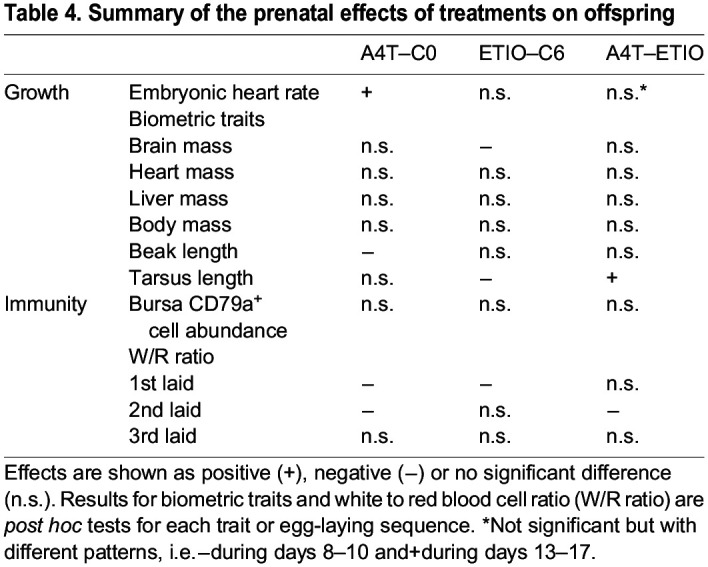
Summary of the prenatal effects of treatments on offspring

In the descriptive part of the study, we found high levels of maternal A4 and testosterone in black-headed gull eggs and a clear increase over the laying sequence, which are in accordance with previous studies of the same (Müller et al., 2004) and other bird species (European starlings: Paitz and Bowden, 2013; Campbell et al., 2020; rock pigeons: Kumar et al., 2018; Wang et al., 2023c). Also, the rapid decrease of A4 and increase of conjugated androgens and ETIO are consistent with these studies. However, unlike what has been observed in other bird embryos, black-headed gull embryos did not show a decrease in testosterone levels within the first 6 days of incubation. One possible explanation for this discrepancy is that the A4 to testosterone ratio in freshly laid black-headed gull eggs (35:1) is very high compared with that in other species (ranging from 17:1 to 22:1). A4 can be converted to testosterone by the early embryos (Bruggeman et al., 2002), and therefore it is possible that such conversion masked the decrease of testosterone in black-headed gull eggs.

As expected, increased A4T in the experiment increased embryonic heart rate in black-headed gulls, which was also found in rock pigeon embryos (Wang et al., 2023a). In this study we did not find an egg-laying sequence effect, which may be due to the fact that the A4T manipulation relative to what was present in the egg was less strong in 2nd and 3rd laid eggs than in 1st laid eggs, as the increase of A4T levels was based on the average and s.d. of 1st laid eggs and endogenous androgen levels steeply increase over the laying sequence. A4 and testosterone were increased by 29.8% and 16.1%, respectively, to their average level in 1st laid eggs but by only 17.1% and 12.9% in 3rd laid eggs (refer to Müller et al., 2004). Contrary to our expectation, increasing ETIO did not increase overall embryonic heart rate, and during day 13–17 of incubation, ETIO embryos had even lower heart rates than A4T embryos. Together with the finding that ETIO embryos had similar heart mass but higher erythrocyte count (Table S6) than A4T embryos, one possible explanation is that more erythrocytes that can carry more oxygen enable ETIO embryos to accomplish the need for metabolism without an increased heart rate. We acknowledge that our study lacks the heart rate data before day 8 of incubation because of methodological limitations, which might be a relevant period when the hormone can affect embryonic heart rate (Wang et al., 2023b).

Although A4T embryos showed increased heart rate, and therefore probably an increased metabolism, this was not reflected in their overall biometric traits (i.e. prenatal growth). This is in line with studies in yellow-legged gulls (*Larus michahellis*) and Chinese painted quail (*Coturnix chinensis*) where increased testosterone did not increase hatchling body mass (Andersson et al., 2004; Parolini et al., 2018). Although one could argue that for heart, liver and body mass the power was below 0.5, effects sizes were low (<0.2). Surprisingly, we found a negative effect of A4T on the beak length of the embryos with strong statistical power (1−β=0.99). Perhaps, A4T embryos would have hatched earlier than control embryos, explaining the lack of elevated growth, but we could not test this as embryos were sampled before hatching. It is worth noting that the statistical power of ETIO treatment on especially beak length was very small (1−β=0.08) so we cannot exclude the possibility that ETIO embryos may have affected beak length but that we were not able to detect this. ETIO treatment had an overall negative effect on embryos' biometric measurements; it decreased tarsus length and brain mass but did not affect hatchling body mass, the latter being consistent with a study in European starling embryos (Campbell et al., 2020). The decrease in tarsus length suggests a negative effect of ETIO on bone growth of which the mechanism is as yet unclear. The decrease in brain mass is also hard to explain as ETIO is well known as a neurosteroid, which has regulatory and protective effects on neuronal cells (Mendell and MacLusky, 2018; Mouton and Duckworth, 2021). Despite a decrease in brain mass, it is still possible that ETIO could improve synaptic connectivity and overall brain performance (Mouton and Duckworth, 2021). Further studies on the behaviour effects of ETIO are therefore essential to test whether such a decrease in brain mass would affect the offspring from a behavioural perspective.

As expected, A4T embryos showed lower W/R ratios than control embryos, yet ETIO embryos did not mimic this effect except in 1st laid eggs. Most vertebrates' stem cell compartments are established during a defined period of embryonic development (Golub and Cumano, 2013), and it is conceivable that testosterone affects early haemopoietic stem cell differentiation, explaining the organizational effects of testosterone on the post-hatching offspring immune system as reported in the literature (Andersson et al., 2004; Müller et al., 2005; Navara et al., 2005; Muriel et al., 2015). Although ETIO was found to promote early embryo erythropoiesis *in vitro*, this was not reflected in the 18 day old embryos (Table S6). This may be because the effects of ETIO on erythropoiesis are only transient (Levere et al., 1967; Irving et al., 1976). It is worth noticing that our hormone manipulations increased the hormone levels in the eggs from different egg-laying sequences to a different extent (see above). Although we do not know the baseline ETIO levels in 2nd and 3rd laid eggs on day 6 of incubation, we may assume that the baseline hormone differences related to the egg-laying sequence (Kumar et al., 2018) can confound the results we observed from our hormone manipulations. Such differences may explain the egg-laying sequence-dependent treatment effects we found in embryo W/R ratio.

We did not find any effect of either A4T or ETIO treatment on the bursa of 18 day old embryos. Earlier studies treating embryos with androgens did find a negative effect on B-cell abundance, but these used much higher, pharmacological doses of the hormone (Oláh et al., 1986; Nagy and Oláh, 2009) whereas our treatment consisted of a physiological dose tailored to the species.

Throughout the study, A4T treatment did not produce the effects that ETIO treatment produced. The possible reason could be that the increase in testosterone and A4 may mask or weaken the effect of ETIO, as the former two are at much higher levels and their effects cost energy that may compromise the energetic demands of ETIO.

In conclusion, contrary to our expectations, increasing ETIO in black-headed gull eggs did not mimic the effects of increasing A4T (Table 4). Our ETIO treatment only showed a negative effect on overall embryo biometric traits but not on the other traits we measured, which raises the question of why the embryos would convert maternal androgen into a metabolite that hampers their physical development. One possible explanation is that given the high maternal androgen levels and therefore high converted ETIO levels in this species, the receptors may already be saturated before our ETIO manipulation. This could account for the lack of effect on embryo size and possibly even the lower heart rate if there was negative feedback occurring. It also remains to be explored whether the embryos would benefit from ETIO in other aspects such as post-hatching behaviours (as discussed above). Despite the presence of ETIO and the evidence that it is converted from testosterone and A4 in eggs of other avian species, as well as its effects in embryo development, our study did not show to what extent maternal A4 and testosterone are converted to ETIO in our species. As we still lack an overview of the androgen metabolic pathways in bird eggs (Wang et al., 2023c), it is likely that are other metabolites could mediate the maternal androgen effects. Other maternal androgen metabolites such as 11-ketotestosterone, 11β-hydroxyandrostenedione, 11β-hydroxytestosterone and 11-keto-dihydrotestosterone are found in mammalian species and, more importantly, they are biologically relevant (du Toit and Swart, 2021; Turcu et al., 2021). Nevertheless, as the early metabolism of maternal A4 and testosterone is due to embryonic activity (Kumar et al., 2019b), this shows that embryos have self-control over the potential effects of maternal hormones, which may indicate the presence of an ongoing mother–offspring conflict over the developmental outcome. Therefore, we call for further studies to find the existence of these metabolites in bird embryos and test the relevance of these metabolites in mediating maternal androgen effects.

## Supplementary Material

10.1242/jexbio.247205_sup1 Supplementary information
