# Supplementary material for: Effects of maternal androgens and their metabolite etiocholanolone on prenatal development in birds
Source: J Exp Biol. 2024 Aug 5;227(15):jeb247205. doi: 10.1242/jeb.247205 (PMC11418167; doi:10.1242/jeb.247205)
Supplement: Supplementary information [file jexbio-227-247205-s1.pdf]

Table S1. Sample sizes of different hormone treatment over years and populations (aviary or wild). Eggs from different laying sequences were collected from the aviary population, displaying in the cells as [total sample size (1st-laid, 2nd-laid, 3rd-laid)]. Only 1st-laid eggs were collected form the wild population. A) For embryonic heart rate measurements; B) For peri-hatching biometric traits measurements; C) For blood W/R ratio measurements; D) For bursa development measurements.

A)

		C0	A4T	C6	ETIO
2020	aviary	28 (9, 7, 12)	48 (11, 14, 13)	9 (2, 4, 3)	31 (6, 12, 13)
	wild	0 (0, 0, 0)	0 (0, 0, 0)	0 (0, 0, 0)	0 (0, 0, 0)
2021	aviary	0 (0, 0, 0)	0 (0, 0, 0)	0 (0, 0, 0)	0 (0, 0, 0)
	wild	11 (11, 0, 0)	15 (15, 0, 0)	5 (5, 0, 0)	9 (9, 0, 0)
2022	aviary	27 (12, 9, 6)	28 (9, 12, 7)	10 (4, 2, 4)	25 (7, 10, 8)
	wild	3 (3, 0, 0)	3(3, 0, 0)	10 (10, 0, 0)	6 (6, 0, 0)
total		69 (35, 16, 18)	94 (38, 26, 20)	34 (21, 6, 7)	71 (28, 22, 21)

B)

		C0	A4T	C6	ETIO
2020	aviary	10 (4, 4, 2)	17 (6, 6, 5)	9 (2, 4, 3)	10 (2, 3, 5)
	wild	0 (0, 0, 0)	0 (0, 0, 0)	0 (0, 0, 0)	0 (0, 0, 0)
2021	aviary	0 (0, 0, 0)	0 (0, 0, 0)	0 (0, 0, 0)	0 (0, 0, 0)
	wild	11 (11, 0, 0)	14 (14, 0, 0)	1 (1, 0, 0)	9 (9, 0 ,0)
2022	aviary	25 (13, 7, 5)	25 (11, 8, 6)	15 (5, 4, 6)	24 (6, 9, 9)
	wild	4 (4, 0 ,0)	4 (4, 0, 0)	8 (8, 0, 0)	9 (9, 0, 0)
total		50 (32, 11, 7)	60 (35, 14, 11)	33 (16, 6, 9)	52 (26, 12, 14)

Journal of Experimental Biology • Supplementary information

C)

		C0	A4T	C6	ETIO
2020	aviary	22 (5, 9, 8)	14 (4, 4, 6)	0 (0, 0, 0)	14 (3, 4, 7)
	wild	0 (0, 0, 0)	0 (0, 0, 0)	0 (0, 0, 0)	0 (0, 0, 0)
2021	aviary	0 (0, 0, 0)	0 (0, 0, 0)	0 (0, 0, 0)	0 (0, 0, 0)
	wild	0 (0, 0, 0)	0 (0, 0, 0)	0 (0, 0, 0)	0 (0, 0, 0)
2022	aviary	14 (10, 2, 2)	13 (6, 4, 3)	10 (2, 2, 6)	14 (4, 6, 4)
	wild	0 (0, 0, 0)	0 (0, 0, 0)	0 (0, 0, 0)	0 (0, 0, 0)
total		36 (15, 11, 10)	27 (10, 8, 9)	10 (2, 2, 6)	28 (7, 10, 11)

D)

		C0	A4T	C6	ETIO
2020	aviary	0 (0, 0, 0)	0 (0, 0, 0)	0 (0, 0, 0)	0 (0, 0, 0)
	wild	0 (0, 0, 0)	0 (0, 0, 0)	0 (0, 0, 0)	0 (0, 0, 0)
2021	aviary	0 (0, 0, 0)	0 (0, 0, 0)	0 (0, 0, 0)	0 (0, 0, 0)
	wild	12 (12, 0, 0)	14 (14, 0, 0)	0 (0, 0, 0)	9 (9, 0, 0)
2022	aviary	0 (0, 0, 0)	0 (0, 0, 0)	0 (0, 0, 0)	0 (0, 0, 0)
	wild	0 (0, 0, 0)	0 (0, 0, 0)	0 (0, 0, 0)	0 (0, 0, 0)
total		12 (12, 0, 0)	14 (14, 0, 0)	0 (0, 0, 0)	9 (9, 0, 0)

Journal of Experimental Biology • Supplementary information

Table S2. Explanations and results of the generalized additive models (GAMs) fitting the non-linear relationship between embryonic heart rate changes and incubation days, with egg sequence, treatment and their interaction either as intercept, curve shape or both.

Model	Formula	Explanation	df	AIC	Delta AIC	Akaike weight
**null**	HR ~ EggT + year + sex + s(x, EggID, bs = "fs") + s(NestID, bs = "re")	Without any predictors	49.18	4797.05	41.61	0.00
**m1**	HR ~ treat + seq + treat*seq + EggT + year + sex + s(x, by = seq, bs = "tp") + s(x, EggID, bs = "fs") + s(NestID, bs = "re")	(See the GAM models explanation above)	58.29	4780.27	27.83	0.00
**m2**	HR ~ treat + seq + treat*seq + EggT + year + sex + s(x, by = treat, bs = "tp") + s(x, EggID, bs = "fs") + s(NestID, bs = "re")	The smooth function set over x by 'treat'; meaning all groups have similar functional responses while variation in responses between two treatment groups is allowed.	55.21	4776.09	23.65	0.09
**m3**	HR ~ treat + seq + treat*seq + EggT + year + sex + s(x, by = treat, bs = "tp") + s(x, by = seq, bs = "tp") + s(x, EggID, bs = "fs") + s(NestID, bs = "re")	The smooth functions set over x were 'treat' and 'seq' respectively, which enables an additive	64.40	4755.21	2.77	0.20

Journal of Experimental Biology • Supplementary information

		effect of each level comparing to 'TreatSeq' in the m9 (see below) which gives interactive effect.				
**m4**	HR ~ treat + seq + EggT + year + sex + s(x, by = seq, bs = "tp") + s(x, EggID, bs = "fs") + s(NestID, bs = "re")	The factor (intercept) 'treat*seq' was dropped comparing to the m1.	57.74	4775.93	23.49	0.00
**m5**	HR ~ treat + seq + EggT + year + sex + s(x, by = treat, bs = "tp") + s(x, EggID, bs = "fs") + s(NestID, bs = "re")	The smooth function set over x by 'treat' comparing to m4.	54.55	4772.86	20.42	0.00
**m6**	HR ~ treat + seq + EggT + year +sex+ s(x, by = treat, bs = "tp") + s(x, by = seq, bs = "tp") + s(x, EggID, bs = "fs") + s(NestID, bs = "re")	The smooth functions set over x were 'treat' and 'seq' respectively, which enables an additive effect of each level comparing to m4.	63.86	4752.44	0.00	0.80
**m7**	HR ~ treat + EggT + year +sex+ s(x, by = treat, bs = "tp") + s(x, EggID, bs = "fs") + s(NestID, bs = "re")	The factor (intercept) 'seq' was dropped comparing to the m5.	54.69	4769.80	17.36	0.00

Journal of Experimental Biology • Supplementary information

**m8**	HR ~ seq + EggT + year +sex+ s(x, by = seq, bs = "tp") + s(x, EggID, bs = "fs") + s(NestID, bs = "re")	The factor (intercept) 'treat' was dropped comparing to the m4.	59.87	4776.79	24.35	0.00
**m9**	HR ~ TreatSeq + EggT + year +sex+ s(x, by = TreatSeq, bs = "tp") + s(x, EggID, bs = "fs") + s(NestID, bs = "re")	'TreatSeq' was created here, which is the interaction between treatment (treat) and egg sequence (seq). TreatSeq has four levels, the control and androgen treatments for both first- and second-laid eggs.	69.64	4780.62	28.18	0.00

Journal of Experimental Biology • Supplementary information

The GAM models explanation

Example model m1: HR ~ treat + seq + treat*seq + EggT + year + sex + s(x, by = seq, bs = “tp”) + s(x, EggID, bs = “fs”) + s(NestID, bs = “re”).

Treat = treatment, seq = egg laying sequence, treat*seq = interaction between treatment and egg laying sequence, EggT = mean-centered eggshell temperature, year = year of experiment, sex = embryo sex, EggID = egg identity and NestID = nest identity. The function *s()* sets up a smooth function over x, separately for each level of the nominal variable indicated by the *by*-parameter which determine the shape of the curve. The variables used as *by*-parameter were included as intercepts. The *bs*-parameter specifies the type of smoothing (“bs” = basis), and in this case is set to “tp”, the default thin plate regression spline. The smooth specification ‘s(x, EggID, bs = “fs”)’ is a factor smooth where non-linear variability from different EggID was taken into account. Finally, the smooth specification ‘s(NestID, bs = “re”)’ was added to depict the effect of random slope from different NestID.

To explore the best fitted model, the set of models (Table S1) were then generated on the basis of the null model by manipulating the composition of fixed factors to include all possible combination of intercepts (i.e., treat*seq, treat and seq) and by manipulating the non-linear variations of HR over x in different levels of the nominal variables (i.e., treat*seq, treat and seq).

Journal of Experimental Biology • Supplementary information

Table S3. Correlations between every embryo biometric measurement. Pearson correlation, r values in the table, all $p < 0.03$ (not shown in the table).

	brain mass	heart mass	liver mass	body mass	beak length	tarsus length
brain mass	1.00	0.36	0.36	0.62	0.36	0.35
heart mass	\	1.00	0.46	0.44	0.24	0.28
liver mass	\	\	1.00	0.47	0.21	0.25
body mass	\	\	\	1.00	0.38	0.44
Head-bill length	\	\	\	\	1.00	0.25
Tarsus length	\	\	\	\	\	1.00

Journal of Experimental Biology • Supplementary information

Table S4. Mean and SD (ng/egg) of hormones of first-laid intact black-headed gull eggs from day 0, day 3 and day 6 of incubation.

hormone	incubation days	mean	SD
	0	47,35	17,65
testosterone	3	57,45	22,42
	6	68,77	52,75
	0	6,97	2,82
conjugated testosterone	3	18,42	10,49
	6	27,68	3,22
	0	2351,05	618,85
Androstenedione	3	1713,40	814,21
	6	616,78	149,33
	0	17,01	7,91
etiocholanolone	3	21,76	10,48
	6	72,59	26,01
	0	5,34	3,26
conjugated etiocholanolone	3	14,54	9,00
	6	68,44	30,07

Table S5. Mean ± SE of embryos' biometric traits in eggs received different treatments respectively. C0 = CLT (vehicle) at day 0, A4T = androstenedione and testosterone treatment at day 0, C6 = CLT at day 6, ETIO = etiocholanolone treatment at day 6.

traits	C0	A4T	C6	ETIO
brain mass (g)	0.407 ± 0.018	0.468 ± 0.019	0.519 ± 0.020	0.438 ± 0.015
heart mass (g)	0.110 ± 0.004	0.106± 0.005	0.116 ± 0.008	0.108 ± 0.005
liver mass (g)	0.203 ± 0.007	0.197 ± 0.007	0.244 ± 0.016	0.216 ± 0.013
body mass (g)	10.32 ± 0.28	10.23 ± 0.31	10.22 ± 0.41	9.51 ± 0.27
beak length (mm)	9.92 ± 0.17	9.35 ± 0.15	9.25 ± 0.16	9.30 ± 0.19
tarsus length (mm)	11.95 ± 0.18	12.34± 0.17	12.12 ± 0.19	11.39 ± 0.17

Table S6. Mean ± SE of erythrocyte and lymphocyte count in eggs received different treatments respectively. CLT = vehicle treatment at day 0 and day 6 of incubation, A4T = androstenedione and testosterone treatment at day 0 of incubation, ETIO = etiocholanolone treatment at day 6 of incubation.

hemogram	treatment	egg laying sequence	mean ± SE
erythrocyte count (10^6/L)	CTL	1st	2.61 ± 0.15
		2nd	2.58 ± 0.18
		3rd	2.99 ± 0.21
	A4T	1st	3.01 ± 0.27
		2nd	2.34 ± 0.16
		3rd	2.41 ± 0.16
	ETIO	1st	3.12 ± 0.31
		2nd	2.99 ± 0.24
		3rd	3.55 ± 0.25
lymphocyte count (10^3/L)	CTL	1st	21.9 ± 0.14
		2nd	15.1 ± 0.18
		3rd	15.3 ± 0.20
	A4T	1st	8.52 ± 0.27
		2nd	7.01 ± 0.16
		3rd	14.3 ± 0.16
	ETIO	1st	9.04 ± 0.31
		2nd	22.1 ± 0.24
		3rd	22.1 ± 0.25

Journal of Experimental Biology • Supplementary information
